# Assessment of natural ventilation strategy to decrease the risk of COVID 19 infection at a rural elementary school

**DOI:** 10.1016/j.heliyon.2023.e18271

**Published:** 2023-07-14

**Authors:** Javier M. Rey-Hernández, Yolanda Arroyo-Gómez, Julio F. San José-Alonso, Francisco J. Rey-Martínez

**Affiliations:** a Department of Mechanical Engineering, Fluid Mechanics and Thermal Engines, Engineering School, University of Málaga (UMa), 29014 Málaga, Spain; b Department of Energy and Fluid Mechanics, School of Engineering (EII), University of Valladolid (UVa), 47002 Valladolid, Spain; c Thermotechnology Consolidated Research Unit (UIC 053), University of Valladolid, Spain; d Energetics Research Group (TEP139), University of Málaga, Spain; e Institute of Advanced Production Technologies (ITAP), Spain

**Keywords:** Natural ventilation, Low-cost ventilation strategy, Covid19, ACH, Decay CO_2_ concentration method, Thermal comfort

## Abstract

Natural ventilation in low-budget elementary schools is the main focus to ensure the health and comfort of its occupants, specifically when looking at the global pandemic related to SARS-COV-2. This paper presents an experimental and novel study of natural ventilation in a public elementary school (Los Zumacales), with a particularly low economic budget. The study was carried out during the winter months of the Covid 19 pandemic. The school is located in the rural area of Castilla y León (North-Western Spain) far from high traffic roads. In this study, a methodology of measuring CO_2_ concentration was applied in nine classrooms in a school. The experimental study shows the level of natural ventilation in each classroom, expressed in Air Changes per Hour (ACH), using the Decay CO_2_ concentration method. The method is proven by comparing the experimental values of the obtained ACH with those determined by the most powerful methods to achieve appropriate ventilation levels. Thus, ensuring health protection protocol in rural schools, against the COVID 19 pandemic. Harvard guide and Spanish regulations (RITE), two widely recognized methods have been used together with the experimentally obtained standard by Rey et al. Only one classroom showed a value lower than 3 indicating poor ventilation. In this study, the degree of thermal comfort in the nine classrooms were also analyzed according to the EN15251 standard. An average indoor temperature of approximately 19 °C was obtained, and the relative humidity was stable and correct according to Spanish regulations. In addition, the risk of infection in each classroom was estimated following the international method recommended by the federation of European Heating, Ventilation, and Air Conditioning Associations (REHVA). The probability of infection in all the cases studied was less than 14%. Therefore, this study provides a strong response against infections illnesses, such as Covid 19, in educational buildings where economic budgets of their facilities are low in both, maintenance and investment.

## Highlights

• Natural ventilation strategy, low-cost strategy, ensuring good health and comfort, by comparing the most important standards.
• SARS-COV-2 has identified the deficiencies in ventilation in educational buildings, leading to risks of disease transmission.
• The monitored study of existing educational buildings allows the identification and design of healthy ventilation strategies.
• To provide the best and most cost-effective strategies for achieving safe indoor air quality in educational buildings.

## 1 Introduction

Since academic lessons and activities have begun again after being interrupted by the pandemic, the risk of Covid-19 infection in classrooms is a major concern. Discussion surrounding safe environments in elementary schools with minimal economic resources to invest in facilities or maintenance is not new topic [1]. It is known that Indoor Air Quality (IAQ) in primary schools is often unhealthy for its occupants [[2], [3], [4]]. Achieving correct IAQ levels is not easy when given inexpensive ventilation strategies, as these strategies are not properly implemented. Additionally, most elementary schools do not have any mechanical ventilation systems to ensure the air renewal, thus natural ventilation is their only ventilation strategy [5,6]. The crisis caused by the pandemic has only served as a reminder of this and, as a consequence, the public debate on proper ventilation in schools has intensified [7,8]. Approximately 75% of Covid-19 infections have been reported with in indoor spaces [[9], [10], [11], [12]]. This data explains, the current concern for adequate ventilation. Considering that the school is a place where the youth population spends many hours (5–8 h/day), a safe indoor environment is one of the basic requirements to ensure the well-being and comfort of students [13,14].

Evidence indicates that, in addition to SARS-CoV-2 being transmitted via large droplets and fomites, it is also transmitted through aerosol inhalation [15,16]. The control of bioaerosols in indoor environments makes it possible to reduce the risk of the virus spreading by air. In order to achieve safe ventilation levels, various methods may be used according to multiple reference guides on how to operate schools during COVID-19. Initially, the use of a mask and hand hygiene, were recommended, and then ventilation was added. Regular ventilation contributes in diluting bioaerosols and decreases their transport, through airflow and adequate air recirculation systems [[17], [18], [19]]. The research of Walkinshaw et al. analyzed air quality in small volume spaces such as aircraft cabins. In their study, volume and ventilation information provided important data that can now be considered when regulation of the required ventilation is needed [20].

Other mechanisms that are used, generally when ventilation is insufficient, are Minimum Efficiency Reporting Value (MERV) 13 [21] and High Efficiency Particle Arresting (HEPA) filters or air purifiers [22,23]. In the study of Amato et al. concluded that the use of air purifiers in school gyms are an effective simple system to guarantee lower exposure of airbone super micron particles in children, where no mechanical ventilation systems are available [24].

Other complementary systems, which have also been used to achieve optimal IAQ and have controlled values are ozonation and photocatalysis equipment [25,26]. They can be considered a quick and safe solution for critical health emergencies in building with a low economic budget, such as public schools.

Indirect indicators are often used to determine viral levels present within the environment. When economic resources are very limited, the best balance of analysis is supported by studying the CO_2_ concentrations. Recently, Zivelonghi et al. reported that tracking CO_2_ levels, in principle could be used for an indirect determination of the potential viral charge in populated conditions and hence for real time estimations of the infection risk function [27]. Jiménez et al. discussed exhaled CO_2_ as a COVID 19 infection risk proxy for different indoor environments and performed a method to calculate the probability of infection when an infected person is present in an enclosed space [28].

A systematic review carried out by Liu et al. stressed the importance of CO_2_ concentration in ventilation control systems and analyzed the development of CO_2_ sensors and control equipment [29]. Many authors evaluate the effectiveness of natural ventilation by analyzing the evolution of the CO_2_ concentration in classrooms. Villanueva et al. studied a strategy based on the manual opening and closing of windows in 19 nursery, primary, and secondary classrooms in the reopening of educational spaces in the Covid-19 pandemic located in a metropolitan area [30]. The results showed that 26% of the classrooms examined had a high concentration of CO_2_ ≥ 700 ppm, in particular secondary school classrooms had the poorest data. On the other hand, they indicate that thermal comfort (Temperature and Relative Humidity) remains at acceptable values in all cases studied. An extensive study carried out by Fernández-Agüera in several schools, showed that in 72% of cases, natural ventilation is insufficient to maintain CO_2_ levels at admissible values and pointed out the urgent need for adequate controlled ventilation system [31]. However, the economic budget of schools can be very limited and may not allow the installation of mechanical ventilation systems. Schibuola et al. evaluated the ventilation system in two schools in Italy, observing that in many cases the concentration of CO_2_ exceeds the allowed values and reflecting that excessive use of natural ventilation could compromise thermal comfort in classrooms, and even produce a waste of energy [32]. In this sense, a study described by Zemitis et al. in a secondary classroom in Latvia showed that the CO_2_ concentration is well above the advisable values when using the manual window opening, and closing system [33]. This indicates that these harmful levels decrease student performance and that such a loaded environment could favor the transmission of infectious diseases. The antecedents presented show that the CO_2_ values in which students are exposed to in classes with manual ventilation are variable and depend on various factors: location of schools, quality of outdoor air and ventilation rate. In this sense, occupant generated CO_2_ as a “natural tracer” gas is widely used to determine ventilation rate [34]. This method is convenient since CO_2_ is inert meaning the sources of emission (people) are in all buildings and there are commercial, cheap and accurate CO_2_ meters.

Another aspect to consider in the use of natural ventilation is that with a well-planned strategy it could contribute to reducing energy consumption with respect to Heating Ventilation and Air Conditioning (HVAC) systems. In this sense Liu et al. proposed a mixed ventilation system, which provides more effective energy savings in temperate climate zones rather than in cold regions, as well as maintaining an indoor comfort thermal environment [35].

In Spain teaching activities were reopened for the 2020-21 academic year with most educational centers only having natural ventilation. However, there was no time to provide adequate ventilation systems and the economic cost of renovation was too high. Faced with this scenario, a pressuring measure was proposed, consisting of the installation of CO_2_ sensors, to determine when it is necessary to renew indoor air in the classrooms by opening doors and windows.

In this study Decay CO_2_ concentration methodology based on a guide about primary schools during the Covid-19 pandemic was developed and applied in nine classrooms of an elementary school located in a rural area of Valladolid (North-Western Spain) [36]. The decay CO_2_ concentration method is applied to determine the ventilation rate, (expressed as Air Change per Hour, ACH). The method was validated by comparing the experimental values of the ACH obtained, with those determined by two theorical standard procedures elaborated by Spanish regulations (RITE) [37] and described in the Harvard guide [38]. Furthermore, the procedure previously described in the literature by Rey et al. [39] was used. The first method, RITE [37], as they are the mandatory ventilation conditions required by the Spanish standard for schools, according to the level of indoor air quality. The second method, Harvard guide [38], as it is an international guide for schools, published during the 2020 pandemic in efforts to minimize the impact of covid in schools, according to the level of ventilation. The third method, Rey et al. [39], as a reference of a validated experimental proposal, carried out using the photoacoustic technique with tracer gases, showing the minimum ventilation flow rate that achieves an optimal level of Indoor Air Quality. Thermal comfort degree of the nine classrooms is also analyzed following the European standard EN 15251 [40].

Finally, the risk of infection, in each classroom was determined by recommended REHVA procedures. According to a respiratory infection risk-based ventilation design method described by Kiil et al. [41] the proposed low-cost ventilation strategy makes it possible to ensure the health of individuals within schools that have limited financial resources to invest in their facilities.

## 2 ‘Case study of “Los Zumacales” elementary school’

For this case study, “Los Zumacales” Elementary School was chosen. It is a public elementary school within the territorial government of Castilla y León, for the education of children from 3 to 12 years old and with surface area of 3800 m^2^. It is located in a small town called Simancas, Valladolid. The school is not directly exposed to streets with high traffic density. The building has 2 floors with 9 classrooms, a library, various offices, bathrooms, a meeting room, a canteen, among other dependencies.

**Fig. 1:**
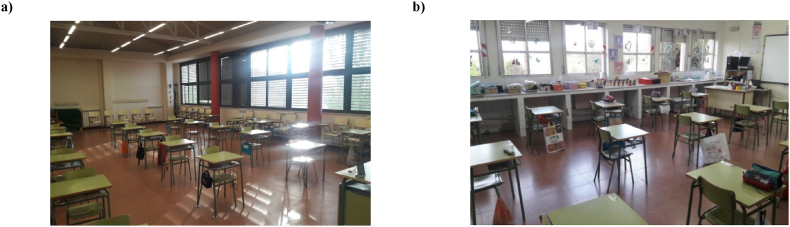
**a) “**5 P” Classroom (10 years-old Students; South-East orientation). **b)** “1I” Classroom (3 years-old Students; South orientation).

All classrooms were studied: 3 classrooms for ages 3-6 years-old and 6 classrooms for children 7-12 years-old classrooms. Fig. 1a and b show two classrooms where the different natural ventilation measures have been carried out. This study was carried out in selected classrooms during the winter months of the 2020–2021 academic year, particularly chosen as the season has the most unfavorable outdoor climate.

CO_2_ meters “Efitest CO_2_ LCD” have been used for measuring and logging CO_2_ (by infrared technology), indoor temperature and RH (model DM72C). The measurement range and accuracy were the following: Range: 0–5000 ppm ±20; Detection temperature: 10 °C to 50 °C ± 0,5; Relative humidity: 20%–85% ± 10; and Sampling time 1.5 s.

## 3 Methodology

Within the nine classrooms included in the study, environmental parameters such as CO_2_, temperature, and relative humidity were monitored to analyze correct ventilation in protecting individual health against Covid-19, as well as the level of thermal comfort. The criteria considered in the analysis of the classrooms studied are summarized in Table 1: Age of students; Orientation; Location of the room in the building (floor); Practices for promoting natural ventilation (number of windows); Occupation (m^2^/student); Number of students per classroom.

**Table 1:** Classroom features.

Classroom (Student age)	Orientation	Floor	Surface (m^2^)	Volume (m^3^)	Number of Windows	Occupation (m^2^/person)	Students
1 I (3)	South	0	59	153	7	8.4	7
2 I (4)	South	0	59	153	6	8.4	7
3 I (5)	South	0	59	153	6	4.9	12
1 P (6)	North	0	59	153	6	5.9	10
2 P (7)	South	1	59	153	6	3.9	15
3 P (8)	North	1	59	153	6	4.2	14
4 P (9)	South	1	59	153	6	5.3	11
5 P (10)	South-East	1	127	507	8	6	21
6 P (11)	North	1	59	153	6	3.9	15

In addition to the level of occupation within the classes, the activity carried out in them was one of the factors that most affects the increase in CO_2_ concentration in the classroom. Thus, the recent compendium [11] of physical activity for children have listed 1.4 1.4 MET (Metabolic activity) for children sitting quietly, studying, taking notes, writing, and having class discussion. Whereas, 1.7 MET was appropriate for a teacher who occasionally stood and walked in the classroom. The activity produced by teachers was not significant since students often dominated the total rate of CO2 generation in classrooms. However, the sum of the emissions from the children and the teacher contributed to the total CO_2_ emission rate. Using a physical activity value of 1.4 MET and averaging across boys and girls, the CO_2_ generation rate in classrooms ranged from 0.147 l/min·person for pre-kindergarten children to 0.343 l/min·person for older children (11 years old).

**Fig. 2:**
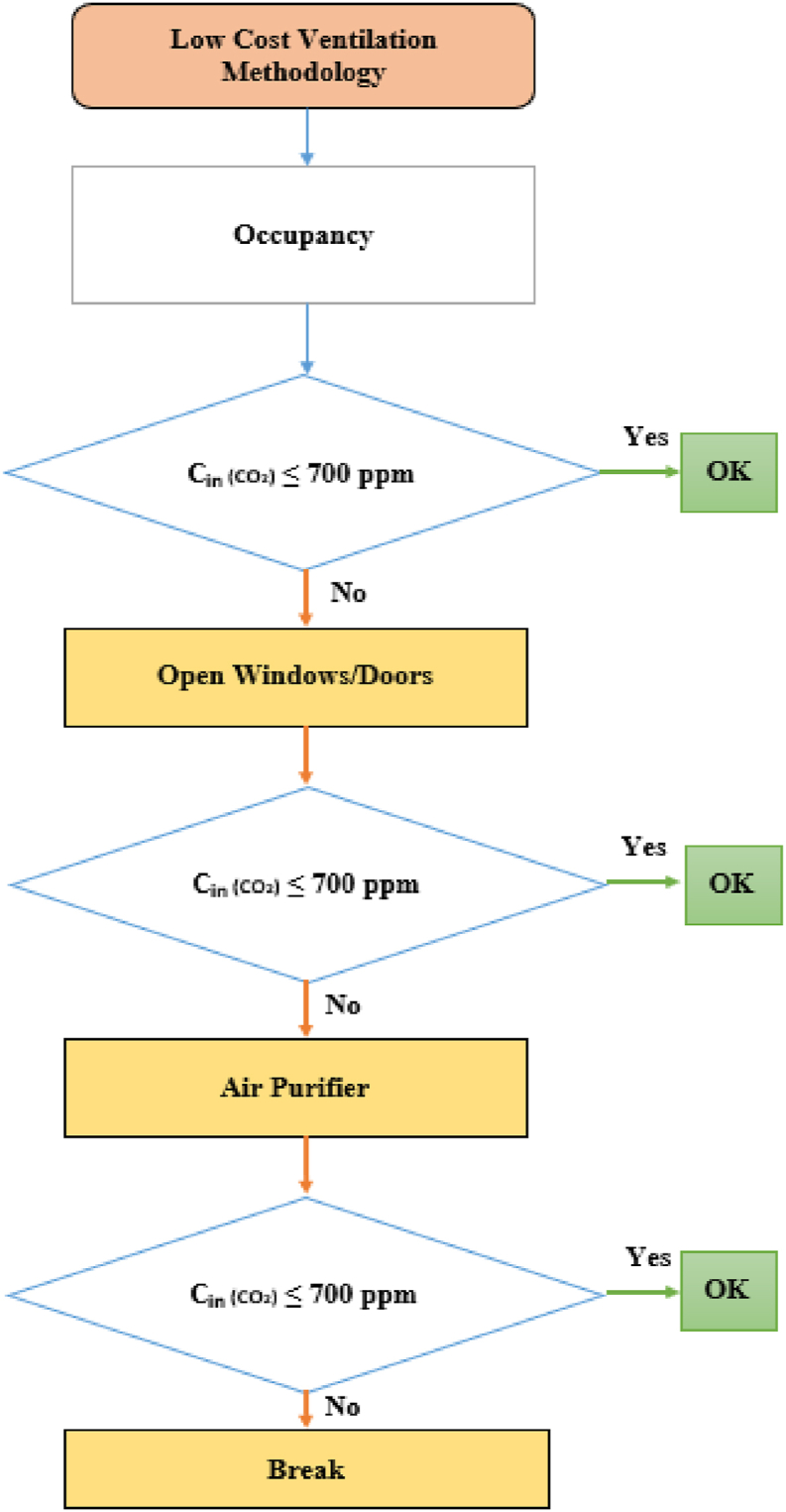
Classroom low-cost ventilation strategy against Covid-19.

The methodology carried out corresponding to the natural ventilation strategy in each classroom is shown in Fig. 2.

In classrooms where natural ventilation is possible, cross ventilation (windows and doors on opposite sides) should be chosen. If natural ventilation is not sufficient, an acceptable ventilation level can generally be achieved by using air cleaning equipment, which must be purified with HEPA filter equipment. The final solution can be a combination of options, for example a combination of air purification and natural ventilation.

The procedure used in this research aimed to evaluate whether natural ventilation is sufficient to achieve the health safety of students and teachers against Covid-19. For this purpose, each classroom was analyzed, and the CO_2_ concentration was monitored. This allowed for the calculation of the ventilation ACH to then be compared with the Spanish regulations (RITE) [37], Harvard guide [38], and proposal of Rey et al. [39].

**Fig. 3:**
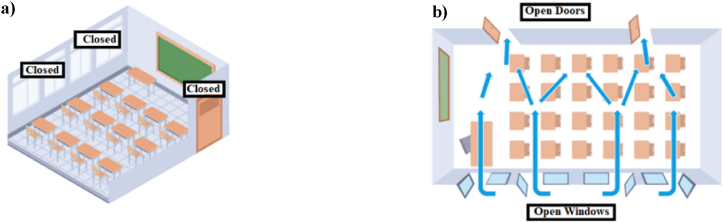
**a)** Doors and Windows Closed in the Classroom. **b)** Doors and Windows Open in the Classroom; Cross Ventilation.

The tests carried out are summarized in Fig. 3. Each day begian with opening the windows without students present, until almost the same concentration of CO_2_ of outside air was achieved (420 ppm). Then, with the classrooms occupied by the students, the windows and doors were closed until the CO_2_ concentration reaches 1000 ppm (Fig. 3a.). Subsequently, doors and windows were opened in a crossover regime (Fig. 3b.), until CO_2_ concentration falls below 700 ppm, which is the national (CSIC) and international (Harvard guide) accepted value for the prevention of the transmission of Covid-19 in closed spaces.

Weather conditions were stable and consistent on sampling days. The wind speed did not exceed 2 m/s in any case and the indoor/outdoor temperature gradient was high due to the fact that these were during winter season.

## 4 Results & discussion

### 4.1 Determination of the CO_2_ concentration at steady state in the classroom

First, the Limited Target Value CO_2_ generation (${LTV}_{{CO}_{2}}$) in the stationary state in each classroom is determined, which will be the limit value that will allow for health protection for the occupants. Once the CO_2_ concentration value is obtained, the first assessment of ventilation level can be determined. To identify the concentration of CO_2_ equation (1) was used [42].

(1) $${{LTV}_{{CO}_{2}}}_{Generation\left(ppm\right)}=Number\;of\;Occupants*\;{CO}_{2\;Exhalation\;Rate\;per\;Occupant}$$

The rate of CO_2_ exhalation per person depends on age, gender, weight, and metabolic activity. Tables published by Persily et al. [43], have been used to determine the CO_2_ generation rate in each case.

Some values of common situations are.

• Students 6–11 years old, seated: 0.0031 l/s = 0.186 l/min per student.
• Adolescents: 0.0044 l/s = 0.264 l/min per Adolescents.
• Teachers (standing and speaking): 0.0061 l/s = 0.366 l/min.

On the other hand, equation (2) allows for the calculation of outside air flow rate using the ACH, and the volume of each classroom [42]. In accordance with the recommendations of the Harvard guide [38], ACH of value 4 is set to ensure good ventilation.

(2) OutsideAirFlowRate(l/min)=ACH*ClassroomVolume

**Fig. 4:**
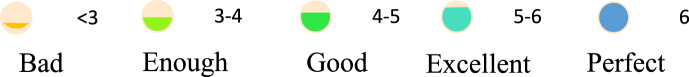
Ventilation level, air changes per hour (ACH), by harvard guide [38].

Based on ventilation in elementary schools, Harvard guide [38] recommends a ventilation level according to ACH greater than 4 for classrooms of 100 m^2^, with 25 students aged 5–8 years (Fig. 4).

These values may change depending on the risk that is assumed. Thus, when there is a lower risk of contagion there is better ventilation. There is no zero-risk situation.

In indoor spaces CO_2_ concentration increases very fast due to the presence of people, as CO_2_ is exhaled when breathing. It was noted that a higher ACH reduced indoor CO_2_ concentrations. This improved the attention level of students, whereas the exposure to high CO_2_ concentrations caused lethargy and often made it difficult for students to pay complete attention impacting the school performance.

Finally, the determination of CO_2_ concentration at steady state in each classroom is calculated, using equation (3) [42].

(3) $${{CO}_{2}}_{Steady-State}\left(ppm\right)=\frac{{LTV}_{{CO}_{2}}\;Generation+Outside\;Air\;Flow\;Rate\;*\;C_{outside\;{CO}_{2}}*1*10^{-6}}{Outside\;Air\;Flow\;Rate*1*10^{-6}}$$

The data obtained by equations are shown in Table 2.

**Table 2:** Data Obtained by CO_2_ Concentration, at Steady State, to Achieve Better Ventilation Levels in each Classroom.

Classroom	Steady-State CO_2_ Concentration (ppm)	Classroom Occupancy (Students + Teacher)	CO_2_ External Average Value (ppm)
1 I	584	8	420
2 I	584	8	420
3 I	675	13	420
1 P	638	11	420
2 P	729	16	420
3 P	711	15	420
4 P	656	12	420
5 P	541	22	420
6 P	729	16	420

The steady-state CO_2_ concentration was variable for each classroom. It was around 620 ppm. However, considering the variations of concentrations throughout the day, it was reasonable to assume a 20% deviation from the target value according to the ventilation guide for classrooms published by Spanish National Research Council (CSIC) [44]. Therefore, a standard value of 700 ppm was considered for each classroom with a very high ventilation level. This data was consistent with what is observed in Table 2. Indoor CO2 levels, and ventilation levels achieved are shown in Table 3 ac-cording to Harvard guide [38].

**Table 3:** Indoor CO_2_ levels (ppm), and ventilation level. Spanish national research council (CSIC).

CO_2_ (ppm)	400–600	600–800	800–1000	>1000
Ventilation	Excellent	Very Good	Good	Bad

To ensure a good ventilation level in each classroom, the goal is to evaluate whether the natural ventilation air flow is correct. Thus, if CO_2_ concentration in the classroom is similar to the calculated steady-state CO_2_ concentration it indicates that the ventilation level is being achieved. Thus, no windows need to be opened.

On the other hand, if CO2 concentration in the classroom was greater than the calculated steady-state CO2 concentration, it meats that the ventilation air renewal objective was not being achieved. Therefore, increasing the flow of outside ventilation air by opening the windows, as shown in Fig. 2, was found to be necessary.

### 4.2 Monitoring design of CO_2_ measurements

The verification of the ventilation and health conditions of teaching spaces, at “Los Zumacales” elementary school, was carried out by CO_2_ meters.

These measurements will provide specific guidelines for the natural ventilation of teaching areas, taking into account the recommendations and health reports. The goal is to ensure maximum safety, along with the best possible thermal comfort.

The measures related to the ventilation of educational spaces are complementary to the prevention measures already implemented: Mandatory use of masks; Reduction of capacity; Maintenance of social distance; Hand washing with hydroalcoholic gel; Disinfection of surfaces; Self-examination of symptoms; Compliance with sanitary isolation measures for people who have been diagnosed as positive, and for their close contacts. It is necessary to comply with these measures.

There are different standards that establish the minimum flow rate to ensure optimal ventilation level in indoor spaces. The main measures to achieve adequate ventilation are in accordance with the Spanish regulations (RITE) [37], which establishes the following IAQ categories (IDA): IDA 1 (day care centers): optimum air quality, 20 l/s·person, equivalent to 72 m^3^/h·student; IDA 2 (classrooms): good air quality, 12.5 l/s·person, equivalent to 45 m^3^/h·person [37]. Spanish legislation does not take into account the transmission of airborne, only IAQ related to the ventilation flow.

Harvard University Guide [38], which focused on educational centers, recommends about 14 l/s person. The required air quality will be achieved by providing a minimum flow of outside ventilation air. This calculation method is used for people with a metabolic activity described by Persily and De Jonge [43] when the production of pollutants from direct human sources is low and when smoking is not allowed.

In spaces and classrooms with natural ventilation, strategies have been directed at reinforcing this ventilation, by opening doors and windows for 10–15 min at the beginning and end of each lesson (morning and afternoon). Moreover, 5–10 min at the end of each hour-lesson.

In this research, the methodology plan has been designed by taking into account the recommendations from the Guide for Ventilation in Classrooms established by the Spanish National Research Council (CSIC-IDEA) [44], as well as the Spanish Ministry of Health (Evaluation of the risk of SARS-CoV-2 transmission).

CO_2_ outdoor concentration was measured every day, before and after measuring CO_2_ indoor concentration. It ranged from 400 ppm to 440 ppm on the most unfavorable day. Therefore, an average value of 420 ppm was assumed for calculated ventilation.

All measurements of indoor CO_2_ concentration were taken in critical points of the classrooms, in accordance with the most recognized international methodologies. A critical point is defined as the area of the classroom that is the most difficult to ventilate according to the prevailing air flows. In practice, this critical point corresponds to an area away from doors and windows, outside the main air flow. The exact spot should be as far away as possible from any person, and at a height of 1.5 m. This makes possible to know the maximum CO_2_ concentration at any given time and provides the most unfavorable case.

Measurements were taken at different times during the school day, in such a way that in each space, there were measurements at 3 different times: At the beginning of the day (9 a.m.); After break (11 a.m.); And at the end of the school day, (1 p.m.).

Occupied classrooms were evaluated, with windows and doors open, using cross ventilation, since this strategy is the most effective (Fig. 3b).

The measurement was carried out under the standard conditions and ventilation of the classrooms. Around 15 min before the ventilation day started, windows and doors were opened until 440 ppm CO_2_ concentration was reached, which means that the classroom was properly ventilated. Then doors and windows were closed until reaching a CO_2_ measurement that exceeded 1000 ppm, this being poor ventilation, the response time before ventilation was measured. The CO_2_ concentration of 1000 ppm is the threshold we adopted as a poor air quality ventilation.

### 4.3 Variation of CO_2_ concentration inside classrooms over time

Ventilation time depends on many factors, which vary from classroom to classroom (volume, occupancy, activity, orientation, outdoor environmental conditions, etc.).

To determine how long it is necessary to keep the windows open, the CO_2_ concentration in the air should be calculated. This value is a positive indicator of the renovation rate of a space when the budget for facilities is very low.

700 ppm is the value set as the calculated steady state CO_2_ reference in each classroom, with 420 ppm being the average outdoor CO_2_ concentration.

**Fig. 5:**
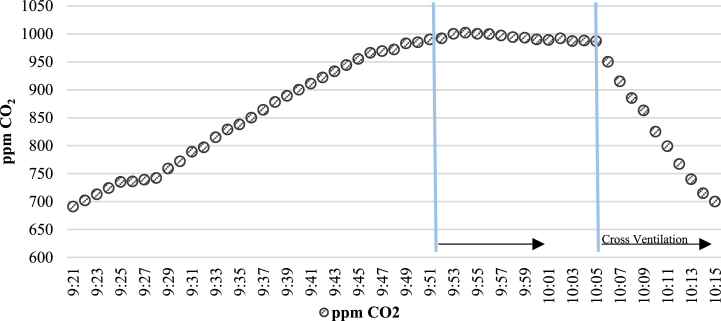
“5 P” Classroom Concentration (ppm CO_2_ vs Time).

Once the classroom was occupied by students, windows and doors of the classroom were closed, observing how the CO_2_ concentration measurements in ppm evolved until reaching 1000 ppm. The 5 P classroom is taken as a model and two natural ventilation systems are evaluated to dilute the excess of CO_2_. The results obtained are shown in Fig. 5.

Fig. 5 shows that opening of doors is not sufficient for the CO_2_ concentration to decrease. On the contrary, the cross ventilation (Fig. 3b, opening doors and windows) leads to a drop in CO_2_ concentration very quickly. In only 10 min, healthy CO_2_ levels for students and teacher are recovered. Considering the CO_2_ variation observed in the 5 P classroom, the natural ventilation method used is to open doors and windows as soon as the CO_2_ concentration reaches 1000 ppm, until the CO_2_ concentration decreases below 700 ppm. This process was repeated three times per day in each classroom during the winter season, obtaining the mean values of the response time as shown in Table 4.

**Table 4:** Measurements of CO_2_ and indoor air temperature obtained.

Classroom	Average Time with Windows and Doors Closed (min) [CO_2_] (1000 ppm)	Average Time with Windows and Doors Open (min) [CO_2_] (700 ppm)	Average CO_2_ (ppm) Outside	Average Classroom Temperature (°C)
1 I	79	13	420	18.5
2 I	92	10	420	19
3 I	44	9	420	19
1 P	51	17	420	19
2 P	41	10	420	20
3 P	38	10	420	20
4 P	53	14	420	19
5 P	98	10	420	19
6 P	30	14	420	20

**Fig. 6:**
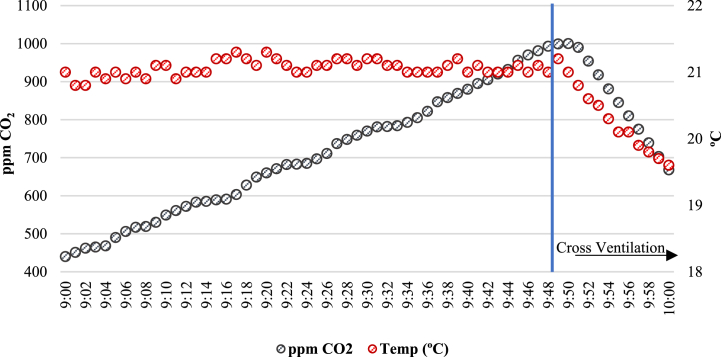
Indoor air temperature and CO_2_ concentration trends in “4 P” classroom.

Variation of CO_2_ Concentration in each classroom have a similar trend to the ones shown in Fig. 6 for the “4 P” classroom.

Fig. 6 shows the variation of temperature and CO_2_ concentration in classroom “4 P”, during the working portion of the day. The performance is similar for all classrooms. When doors and windows are opened, the decrease in CO_2_ concentration is much more intense than the decrease in temperature.

In the 10 min necessary until CO_2_ recovers to an acceptable level (<700 ppm) the temperature drops less than 2 °C. In all the classrooms studied, the temperature remains slightly above 19 °C. Therefore, the CO_2_ renewal rate is much higher than the thermal load loss in the classroom.

### 4.4 ACH by natural ventilation in each classroom

Measuring ventilation in each classroom by ACH is a very accurate methodology. One ACH means that, in 1 h, a volume of outside air enters the room equal to the volume of the room. Thus, due to the continuous mixing of air, this results in 63% of the inside air being replaced by outside air. With 2 ACH in the room, 86% is replaced, and with 3 ACH, 95%.

To determine ACH ventilation, Equation (4) has been used [42].

(4) $$ACH=\frac{-1*\ln\left(\frac{C_{1}-C_{outside}}{C_{0}-C_{outside}}\right)}{t_{1}-t_{0}}$$

On the other hand, the theoretical ACH are determined, based on the minimum flow rate established by RITE (12.5 l/s·person), Harvard guide (14 l/s·person) and Rey method (17 l/s·person). Equation (5) has been used [42].

(5) ACH=(ls·person)*(numberofpeople)*(3600sh)*(0.001m3l)volume(m3)

Opening windows and doors can potentially lead to acoustic issues. It is necessary to reach a balance between health risk, thermal comfort, and acoustic discomfort or difficulties. Reducing noise in corridors is preferred, rather than closing doors.

The opening of windows with the consequent flow of external air can lead to increased levels of pollutants from outside in highly polluted areas. In this case study, due to the school being located in a rural area, the external air quality remained.

Table 5 shows ACH values obtained from ventilation in each classroom. The first column shows the values calculated by means of the experimental measurements of CO_2_ (Equation (5)). The second column shows the values obtained and the minimum theoretical ventilation flow proposed by the Spanish regulations (RITE) to achieve an IDA 2 ventilation level [37]. The third column shows minimum ACH values to achieve a favorable ventilation, with the theoretical ventilation air flow proposed by Harvard Guide for elementary schools [38]. Finally, the fourth column shows ACH values with the flow rates of air proposed in the experimental study by Rey et al. [39] to achieve an adequate ventilation level.

**Table 5:** ACH values obtained per classroom.

Classroom	ACH (h-1)	ACH(h-1)	ACH(h-1)	ACH (h-1)
	Experimental measurements	RITE	Harvard guide	Rey et al.
1 I	3.4	2.4	2.6	3.2
2 I	4.4	2.4	2.6	3.2
3 I	4.9	3.8	4.2	5.2
1 P	2.6	3.2	3.6	4.4
2 P	4.4	4.7	5.3	6.4
3 P	4.4	4.4	4.9	6
4 P	3.1	3.8	4	4.8
5 P	4.4	2	2.2	2.7
6 P	3.1	4.7	5.3	6.4

**Fig. 7:**
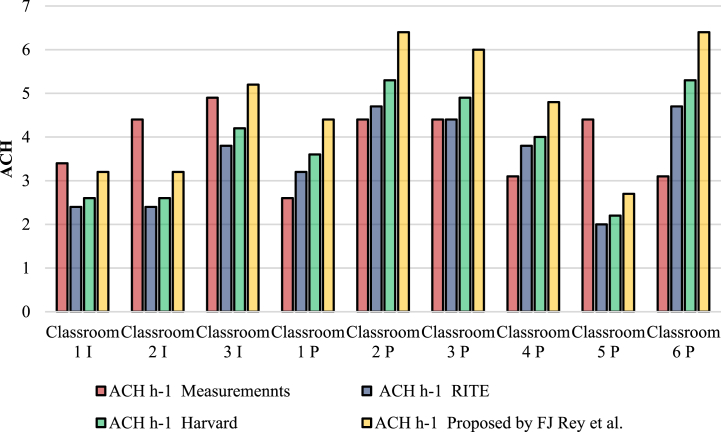
ACH (h-1) Ventilation in each Classroom Measured Experimentally Versus Minimum Proposed by the Spanish regulations (RITE), Harvard Guide, and Experimental Study by Rey et al. to achieve an adequate Ventilation Level.

ACH calculated from all the methods used, are shown in Fig. 7.

Comparing ACH natural ventilation value (h^−1^) in each classroom, it is shown that its amount is between 4 and 5 air changes per hour in 5 classrooms (2I, 3I, 2 P, 3 P and 5 P). This is what is recommended by Harvard guide [1] (Fig. 7) as a favorable ventilation level. Values bigger than 3 ACH, are achieved in 3 classrooms (”1I”, “4 P” and “6 P”). This being a suitable level of ventilation, and only the classroom (1I) complies with the recommended values IDA2 in the Spanish regulations (RITE). Values less than 3 ACH are obtained in a single classroom “1 P”, thus displaying a poor ventilation level and not fulfilling the recommended values IDA2 in the Spanish regulations (RITE) [37].

Analyzing ACH values measured for each classroom and comparing these with Spanish regulations (RITE) [37], Harvard guide [38], and Rey et al. [39], it can be noted that only “1I”, “2I”, “3I” and “5 P” classrooms, are above the recommended values.

It is confirmed that the value of natural ventilation ACH (h^−1^) obtained for classrooms “1 P”, “4 P” and “6 P” do not comply with the recommended Spanish regulations (RITE) [37] or Harvard Guide [38]. Only classroom “1 P” shows a value lower than 3, which is considered a poor ventilation level.

The majority of the “P” classrooms have an ACH below the standard, except for class “5 P”, which the ACH value is higher. This is due to the high volume within this particular classroom. Classrooms “2 P” and “3 P″comply with the value of the Spanish regulations (RITE) [37].

To improve ventilation levels, controlling the degree of thermal comfort, and minimizing energy expenses, it would be necessary to renovate the classrooms using a mechanical ventilation system. However, economic or installation issues hinder the retrofitting of this controlled and mechanical ventilation system.

### 4.5 Natural ventilation supported by air cleaning equipment

In classrooms “1 P”, “4 P” and “6 P”, with ACH values lower than the recommended values IDA 2 in the Spanish regulations (RITE) [37], a proposal of installing clear air equipment should be considered. According to the methodology described in Fig. 2. This system would help to increase the ventilation level through natural ventilation strategies. In addition, this solution requires the use of a filter system, HEPA filter or other filters that are similar. This would contribute to cleaner air by removing solid particles and aerosols where Covid-19 or another virus can be deposited.

ACH_objective_ of natural ventilation supported with an air cleaner equipment, in classrooms “1 P”, “4 P”, and “6 P” has been determined by Equation (6). This makes it possible to achieve a ventilation level greater than the recommended values of IDA 2 in the Spanish Regulations (RITE) [37]. The air renewal achieved by different means in the same room at the same time are additive:

(6) $${ACH}_{objetive}={ACH}_{ventilation}+{ACH}_{Cleaning\;air\;systems}$$

Clean Air Delivery Rate (CADR), for a required ACH cleaning air system is obtained by Equation (7):

(7) $$CADR={ACH}_{cleaning\;air\;systems}*\;Classroom\;Volume$$

The limited economic resources to invest in the facilities leads to the possibility that more than one cleaning air system can be used until the required flow rate is achieved. If it is possible, the purifier should be placed in the center of the classroom and should not blow directly at its occupants.

The most effective system is filtration. Filtration consists of filtering the polluted air through a high-performance filter, usually a HEPA (High Efficiency Particulate Air) filter. This filter retains particles, which then provide clean air. HEPA H13 or higher (>99.95% efficiency) is recommended.

Air cleaning systems are introduced in classrooms (1 P, 4 P and 6 P) with less natural ventilation, and poor ventilation levels in order to achieve the ACH value of 5, according to Spanish regulations [37], and Harvard guide [38]. In this way, it is possible to protect occupants' health against Covid-19.

The required CADR of this purifier for the volume of the classroom would be 306 m^3^/h (Equation (7)). Table 6 shows the calculated results of the three classrooms with cleaning air systems.

**Table 6:** ACH values obtained in classrooms with natural ventilation and air purifiers.

Classroom	ACH h-1 Measurements	ACH h^−1^ RITE	ACH h^−1^ Harvard guide	ACH Objective h^−1^	ACH h^−1^ Cleaning Air System	CADR (m^3^/h)
1 P	2.6	3.2	3.6	5	2	306
4 P	3.1	3.8	4	5	2	306
6 P	3.1	4.7	5.3	5	2	306

### 4.6 Classroom thermal comfort

**Fig. 8:**
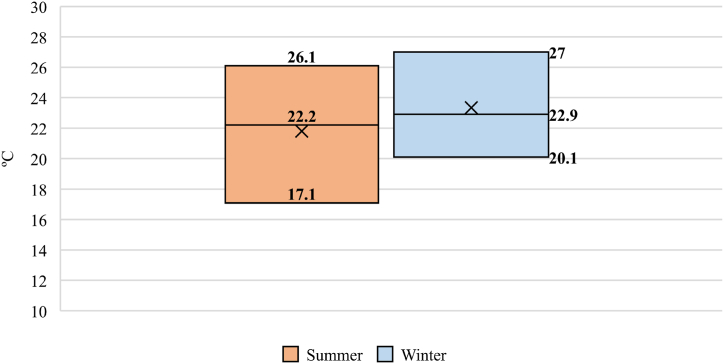
Thermal Comfort Level for Schools by Peixian Lia et al. [19].

According to EN 15251 and Bragger et al. [40,45] as shown in Fig. 8, to define acceptable temperature ranges in buildings the minimum level of thermal comfort in winter for schools in Europe is 20.1 °C. However, the measured temperature data in each classroom indicates that in order to achieve natural ventilation with an appropriate IAQ level and an ACH value around 5, thermal comfort may be slightly outside of the range.

**Fig. 9:**
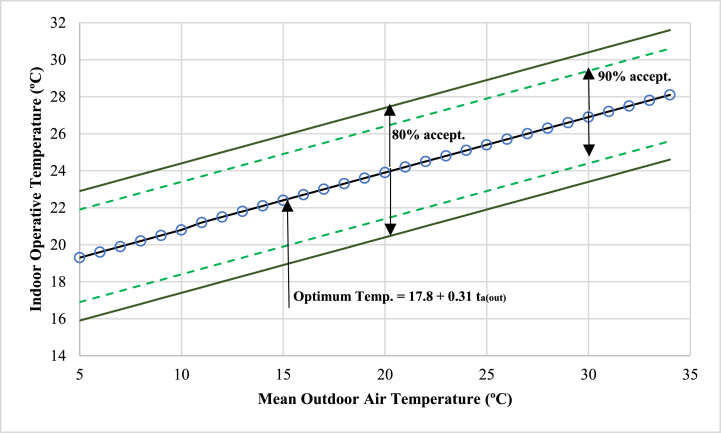
Thermal acceptance of occupants [44].

If the ventilation system of each classroom is considered to be natural the thermal comfort can also be described using the adaptive method according to Dear et al. [46]. Fig. 9 shows that outside temperature is simply an arithmetic average of the mean monthly minimum and maximum daily outdoor air temperatures for the period analyzed.

During the measurement period, the average outdoor temperature ranged between 8 °C to 12 °C, and the average air temperature was between 19 °C to 20 °C. Comparing with the values of the graph proposed by Dear et al. [46] (Fig. 9). It is observed the indoor operating comfort temperature is out of range, but above 90% of the occupants' acceptance.

An important aspect related to the ventilation levels in each classroom is the thermal comfort. Each classroom is heated by an all-water system through radiant heat emitters, and a boiler fueled by diesel.

An average indoor air temperature measured in the classrooms had been between 19 °C to 21 °C, oscillating throughout the day in each classroom.

The relative humidity measured has remained stable, 40%–60%, within the values regulated by Spanish regulations (RITE) [37].

To maintain this comfort, the Heating System is on, even with open windows and doors. Under this condition, diesel consumption increased by 50% compared to the average for the last five years.

### 4.7 Assessment of the risk of infection by COVID 19 or other respiratory diseases

The amount of infectious virus inhaled by a person determines the probability of infection. CO_2_ is exhaled along with aerosols from infected individuals so it can be used as an indirect indicator of the concentration of such viruses [28]. Therefore, keeping CO_2_ as low as possible in an enclosed area allows for optimizing the protection provided by ventilation. The internationally recognized model developed by Jimenez et al. allows for the ability to determine the probability of the transmission of COVID 19 or other infectious diseases by aerosols [28].

The amount of infectious virus inhaled by a person significantly influences their likelihood of becoming infected due to several key factors related to the interaction between the virus and the individual.

The viral dose, known as the amount of virus to which a person is exposed, plays a fundamental role in infection rate. The higher the viral dose that enters the body, the greater the chances of the virus being able to establish and replicate within the cells of the organism. A sufficiently high viral dose can overcome the defense barriers of the immune system and enable successful virus propagation. Conversely, a low viral dose may not be sufficient to overcome immune defenses, thus resulting in a lower probability of infection.

Furthermore, the quantity of inhaled virus is also related to viral replication capacity. The more viral particles present in the inhaled air, the greater the availability of virus to infect the cells of the organism. A higher quantity of viral particles increases the chances of some of them successfully entering cells and triggering the infection process. As more cells become infected and the virus replicates, the viral load in the organism increases, which in turn, can lead to a more severe illness.

Moreover, the response of the immune system is also affected by the quantity of inhaled virus. When exposed to a greater quantity of virus, the immune system may face a higher burden and require more time to mount an effective response. If the quantity of virus is overwhelming, the immune system may take longer to identify and combat all viral particles, thereby increasing the probability of the virus establishing itself and causing an infection.

The quantity of infectious virus inhaled by a person influences their probability of becoming infected due to the viral dose, viral replication capacity, and the response of the immune system. A higher quantity of virus increases the probability of the virus establishing, replicating, and evading the body's defenses, resulting in a greater likelihood of infection. Therefore, reducing exposure to high concentrations of viruses using preventive and control measures, is essential in decreasing the probability of infection.

Measuring CO_2_ levels is a perfect tool for controlling COVID-19 transmission. Elevated CO_2_ indicates poor ventilation, leading to higher concentrations of viral particles in aerosols and increased transmission risk. Monitoring CO_2_ helps assess transmission risks and implement measures to improve ventilation.

Maintaining low CO_2_ levels optimizes ventilation's protective benefits. Adequate ventilation dilutes and removes viral aerosols, reducing infection probability. CO_2_ measurements identify poorly ventilated areas, enabling corrective actions like increased air exchange or air purifiers to enhance circulation and minimize transmission risk.

CO_2_ data provides quantitative information for decision-making. Monitoring CO_2_ helps adjust occupancy, implement safe measures, and improve ventilation in indoor spaces. Using CO_2_ measurements guides effective preventive actions, reducing COVID-19 infections and safeguarding public health.

Measuring CO_2_ levels plays a significant role in controlling COVID-19 transmission. It informs air quality, ventilation effectiveness, and transmission risks. Maintaining low CO_2_ levels through adequate ventilation reduces infection risk. Utilizing CO_2_ data in decision-making and preventive measures is crucial for public health and limiting viral spread.

In this paper, this procedure is applied to estimate the probability of infection and the number of infected students in each classroom, under two assumptions: an infected teacher or an infected student. The model assumes that the aerosols are homogeneously distributed in the room, does not consider contact transmission, and a minimum distance of 2 m is maintained.

It has been considered that all occupants wear masks with an efficiency of 50% for the emission and 30% for the reception of aerosols. It is also considered that the occupants spend a time of 50 min in the classroom per lecture. Two situations are assumed a) the teacher is infected or b) a student is infected. The simulation results are shown in Table 7.

**Table 7:** Probability of infection and number of infected people per classroom.

Classroom (Age)	1. Teacher Infected		2. Student Infected		
	1. Probability of infection (Students) (%)	1. Number of infected people	2. Probability of infection (Teacher) (%)	2. Probability of infection (Students) (%)	2. Number of infected people
1 I (3)	10.79	1.62	7.11	9.81	1.54
2 I (4)	7.22	0.51	4.73	6.55	0.51
3 I (5)	6.4	0.77	4.19	5.81	0.74
1 P (6)	13.32	2	8.83	12.14	1.91
2 P (7)	7.22	1.08	4.73	6.55	1.03
3 P (8)	7.22	1.01	4.73	6.55	0.98
4 P (9)	10.79	1.19	7.11	9.81	1.15
5 P (10)	2.23	0.47	1.45	2.02	0.45
6 P (11)	11.21	1.68	7.4	10.21	1.6

**Fig. 10:**
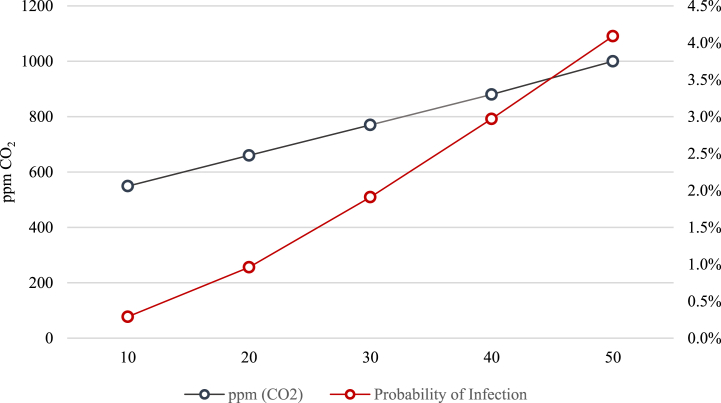
Trends of CO_2_ concentration and probability of infection.

Fig. 10 shows the probability of infection from 10 min to 50 min with classroom occupancy in “4 P” Classroom. At 50 min CO_2_ concentration starts to decrease. This is due to the end of the lecture and upon observing that the risk rises, the windows are opened.

It is observed that the CO_2_ control is valid for periods of time less than 45 min.

However, once 45 min are exceeded, the probability of infection increases more than the CO_2_ concentration, so the CO_2_ control is only efficient in preventing infection when the class has a maximum of 45 min. This evidence shows that it is necessary to open windows/doors at the end of each lecture.

The results obtained show that with the proposed ventilation system, no student would be infected. This includes hypothetical cases that even if the teacher or a student were infected, they do not offer a substantial risk as a source of contagion.

## 5 Conclusions

Ensuring positive natural ventilation in the classroom through an adequate level of ACH protects a reduction in infection of Covid-19. In addition, it favors attention and school performance, since exposure to high concentrations of CO_2_ produces lethargy and makes paying attention difficult.

Most schools do not have the financial budget to upgrade their facilities and ventilation equipment to cope with the Covid 19 pandemic. Therefore, low-cost natural ventilation strategies that are properly implemented can help ensure the health of occupants in elementary schools.

The steady-state concentration of CO_2_ for each classroom will depend on the generation of CO_2_ pertaining to the presence of students and teacher as well as the activity (met). Outside, the CO_2_ concentration measurement was positive, 420 ppm, and the outdoor air flow, which it has been assumed, is the one recommended in the Harvard guide for schools (14 l/s person).

The values obtained are different for each classroom, but considering an error of less than 14%, a standard value for all of them can be set (700 ppm). This value is recommended according to Spanish regulations.

Not all classrooms achieved correct ventilation level, due to lower cross-ventilation airflow, either because of their orientation, higher occupancy, or increased student activity.

However, due to economic limits in the budget, it was proposed a simple and low-cost solution that improves and protects a positive level of ventilation in compliance with the Spanish regulation (RITE).

In the three classrooms that did not reach an optimal ventilation level, the introduction of air cleaning systems is proposed, calculating ACH and CADR, 306 m^3^/h of clean air supply. Therefore, ACH_objetive_ value of 2 in cleaning air systems.

The degree of thermal comfort in the classrooms is analyzed too, following the adaptive model according to EN 15251 due to the ventilation system is natural. The minimum temperature for thermal comfort in winter is 20 °C. Therefore, although thermal comfort is not achieved for any of the classrooms, the average of the measurements obtained for the interior temperature for each classroom are close to the minimum temperature. Therefore, thermal comfort operating temperature corresponding to an acceptance by the occupants of 90%. Furthermore, it has been possible to verify that the probability of infection in the classroom is less than 5% in all the cases studied.

The consumption of fuel oil in the heating system has increased by 50% more than the average of the previous five years. However, it is worth the economic cost if the health and performance of teachers and students are improved.

In this scenario and in a post Covid-19 period to achieve an excellent ventilation level in elementary and primary schools, it is proposed to increase air ventilation to 14 l/s person. Low-cost ventilation strategies, based on natural ventilation, help to maintain a safe environment as long as they are implemented correctly.

## Author contribution statement

Javier M Rey-Hernandez; Francisco J. Rey-Martinez: Conceived and designed the experiments; Performed the experiments; Analyzed and interpreted the data; Contributed reagents, materials, analysis tools or data; Wrote the paper.

Julio F. San Jose-Alonso; Yolanda Arroyo: Performed the experiments; Analyzed and interpreted the data; Contributed reagents, materials, analysis tools or data; Wrote the paper.

## Data availability statement

The data that has been used is confidential.

## Declaration of competing interest

The authors declare that they have no known competing financial interests or personal relationships that could have appeared to influence the work reported in this paper
